# Two new species of *Hygrophorus* from temperate Himalayan Oak forests of Pakistan

**DOI:** 10.3897/mycokeys.56.30280

**Published:** 2019-07-10

**Authors:** Arooj Naseer, Abdul Nasir Khalid, Rosanne Healy, Matthew E. Smith

**Affiliations:** 1 Centre for Undergraduate Studies, University of the Punjab, Lahore, 54590, Pakistan; 2 Department of Botany, University of the Punjab, Lahore, 54590, Pakistan; 3 Department of Plant Pathology, University of Florida, Gainesville, FL, USA

**Keywords:** Biodiversity, Community structure, Dir, ECM, Shawar Valley

## Abstract

The genus *Hygrophorus* is poorly studied from Asia. From Pakistan, only one species has been reported so far. Two new species in the genus have been collected from Himalayan oak forests of Pakistan. *Hygrophorusalboflavescens* (section Pudorini, subgenus Colorati) is characterised by its pure white, centrally depressed pileus, occurrence of white stipe with yellow patches at lower half and broader (4.98 μm) basidiospores. *Hygrophorusscabrellus* (section Hygrophorus, subgenus Hygrophorus) is characterised by its yellowish-green stipe with white apex that has fine scales on the entire stipe, an off-white pileus with dark green and greyish fibrils, ovoid to ellipsoid basidiospores and clavate 4-spored basidia. Macro- and micromorphological descriptions have revealed that both these taxa are not yet described. Phylogenetic estimation based on DNA sequences from the internal transcribed spacer (ITS) region and large subunit (LSU) of the nuclear ribosomal DNA (rDNA) genes, is congruent with the morphological characters that help to delimit these as new species of *Hygrophorus*. Allied taxa are also compared.

## Introduction

The genus *Hygrophorus* Fr. (Hygrophoraceae, Agaricales) is one of the ectomycorrhizal (ECM) genera in Agaricales. The genus name *Hygrophorus* Fr. (Hygrophoraceae, Agaricales) comes from *hygro* meaning moisture and *phorus* meaning bearer. This may refer to the glutinous to viscid pileus character that many of these fungi have due to a layer of gel that makes them sticky to touch when moist. The genus is characterised by diverse basidiomata colours, basidiomata which are tricholomatoid, collybioid, clitocyboid or omphalinoid, lamellae that are subdecurrent, spores that are smooth and hyaline and a hymenium without cystidia. Basidiomata in this group vary from small to large; thin to fleshy; dry to very glutinous or viscid pileus; with a dry to glutinous, glabrous or fibrillose, generally pruinose or granulose stipe (Singer 1986; Bas et al. 1990; Boertmann 1995; Young 2005; Kovalenko 2012). Colour of the pileus is a characteristic feature in the classification of *Hygrophorus* especially at the level of subsection (Hesler and Smith 1963). Sect. Hygrophorus has white to cream basidiomata while taxa with colourful basidiomata are in different sections and subsections (Fries 1874; Singer 1943; Candusso 1997).

The family Hygrophoraceae Lotsy was revised by Lodge et al. (2014) on the basis of integrated molecular phylogeny, morphological analyses, pigment chemistry and ecology. They classified the family with three new subfamilies, eight tribes, eight subgenera, 26 sections and 14 subsections. SubgenusColorati of genus *Hygrophorus* contain coloured mushrooms. In the new classification, the subg. Colorati (Bataille) E. Larss. has been divided into three sections: *Olivaceoumbrini* (Bataille) Konrad & Maubl., *Pudorini* (Bataille) Konrad & Maubl. and *Aurei* (Bataille) E. Larss. In addition, the section Pudorini is divided into two subsections: *Clitocyboides* and *Pudorini*. The subgenusHygrophorus is divided into two sections: *Hygrophorus* and *Fulventes*.

*Hygrophorus* species are globally distributed and mostly occur in woodlands and forests with pines or with ectomycorrhizal (ECM) angiosperms (Bas et al. 1990). *Hygrophorus* are essential components of ECM communities of temperate regions in the Northern Hemisphere (Tedersoo et al. 2010). Recently, a new edible species, *H.parvirussula* has been described from south-western China (Huang et al. 2018) and it belongs to HygrophorussectionPudorini. A few studies on the genus have been performed in Pakistan. Only one species, *Hygrophoruschrysodon*, was reported as a new record by Razaq et al. (2014), from the western Himalayan forests of Pakistan. Here we present two new species of *Hygrophorus* based on both morphology and molecular phylogeny.

## Materials and methods

### Morpho-anatomical analyses

Collections were made during field investigations for ECM communities associated with the oaks of Swat and Dir districts, Khyber Pakhtunkhwa province, Pakistan during 2014–2016. Basidiomata were found in a pure *Quercus* forest from Shawar Valley, Swat that is representative of the western Himalayan Province (Naseer et al. 2017b) and from Toa, Alpurai forests (Naseer et al. 2017), Swat, KP, Pakistan. The ECM roots were collected from the same forests as well as Biar, Upper Dir, KP, Pakistan. Biar is located in moist parts of dry temperate zones and it has *Q.baloot* as the leading species (89.44%) with *Q.dilatata* (10.46%). Basidiomata were collected following Lodge et al. (2014) and photographed in their natural habitats using a Nikon D70S camera. Morphological characters were recorded from fresh specimens. Colours were designated with reference to mColorMeter application (Yanmei He, Mac App Store). Specimens were deposited in the Herbarium, Department of Botany, University of the Punjab, Lahore, Pakistan (LAH) and the University of Florida Fungal Herbarium, Gainesville FL, USA (FLAS).

Microscopic characters are based on freehand sections from fresh and dried specimens mounted in 5% (w/v) aqueous potassium hydroxide (KOH) solution. Tissues from lamellae, pileipellis and stipitipellis were mounted in phloxine (1%) for better contrast and examined using a Meiji Techno MX4300H compound microscope.

A total of thirty basidiospores, basidia, cystidia and hyphae were measured from each collection. For basidiospores, the abbreviation “*n/m/p*” indicates *n* basidiospores measured from *m* fruit bodies of *p* collections. Dimensions for basidiospores are given using length × width (L × W) and extreme values are given in parentheses. The range contains a minimum of 90% of the values. Measurements include arithmetic mean of spore length and width for all spores measured, Q means spore length divided by spore width and avQ indicates average Q of all spores ± standard deviation.

### Molecular analyses

Genomic DNA was extracted from basidioma gills following a modified CTAB extraction method (Bruns 1995) and from ectomycorrhizal roots by Extract-N-AmpTM Kit (Sigma- Aldrich, St Louis, MO, USA). ITS and LSU regions of nuclear rDNA were amplified using the pairs of primers ITS1F-ITS4B and LR0R-LR5 (Vilgalys and Hester 1990, White et al. 1990, Gardes and Bruns 1993). Polymerase chain reactions (PCR) were performed in 25 μl volume reactions. Visualisation of PCR products were accomplished using SYBR Green and 1.5% agarose gels with TAE buffer for gel electrophoresis. Successful amplicons were purified by enzymatic purification using Exonuclease I and Shrimp Alkaline Phosphatase enzymes (Werle et al. 1994). Purified products were sequenced by the University of Florida’s Interdisciplinary Center for Biotechnology Research (http://www.biotech.ufl.edu/). Sequence chromatograms were trimmed, edited and assembled using Sequencher 4.1 (GeneCodes, Ann Arbor, MI). Once sequences were assembled and edited, they were deposited in GenBank (http://www.ncbi.nlm.nih.gov).

Consensus sequences for ITS and LSU were used to query GenBank and the UNITE database using BLAST searches. Representative sequences from across the genus *Hygrophorus* were downloaded and imported into an alignment in Bioedit (Hall 1999). Sequences from ECM roots were also included in the ITS alignment. *Cantharocybegruberi* (JN006422, DQ200927) sequences were chosen to root the phylogenetic trees following Razaq et al. (2014). Sequences were aligned with the programme MUSCLE (Edgar 2004). Maximum likelihood analyses for individual gene regions were performed via CIPRES Science Gateway (Miller et al. 2010) employing RAxML-HPC v.8. Rapid bootstrap analysis for the best-scoring ML tree was configured for each dataset. For the bootstrapping phase, the GTRCAT model was selected. One thousand rapid bootstrap replicates were run. A bootstrap proportion of ≥ 70% was considered significant.

## Results

### Molecular phylogenetic analyses

Consensus sequences for the ITS region of *H.alboflavescens* were 601–638 bp after trimming. BLAST searches in NCBI and UNITE revealed 91% similarity to *Hygrophoruspenarioides* Jacobsson & Larss. (EF395370, EF395371, EF395372 & UDBO1556) from Sweden (99% query cover, 0.0 E value). The two ITS sequences from ECM root tips of *Q.incana* from same forest (Shawar Valley) matched with *Hygrophorusalboflavescens* fruiting body sequences and these are depicted in the phylogenetic tree (Fig. 5).

The consensus sequence for the LSU region of *H.alboflavescens* was 780 bp after trimming. Initial BLAST analysis revealed it as 94% similar to *H.sordidus* Peck. (AF042562) from the USA and *H.russula* (Schaeff. ex Fr.) Kauffman, (AY586663) from Sweden (100% query, 0.0E value).

The ITS analysis revealed that sequences from *Hygrophorusalboflavescens* clustered with *H.penarioides* and *H.sordidus* with moderate bootstrap support within section PudoriniofsubgenusColorati. The LSU based phylogram showed that *H.alboflavescens* clustered with *H.sordidus* (Fig. 5). LSU sequences for *H.penarioides* were not available.

The consensus sequences for the ITS region of *Hygrophorusscabrellus* nom. prov. were 603–604 bp. BLAST results revealed that these sequences were 89% similar to *Hygrophoruseburneus* (Bull.) Fr. (AY463485, AY463484 & AY242855) with 100% query coverage. The consensus sequences also showed 87% similarity to *H.cossus* (Sowerby) Fr. as *H.quercetorum* P.D. Orton, which has been synonymised with *H.cossus* (Larsson and Jacobsson 2004) (AY463489) and *H.cossus* (AY242852) from Sweden with 100% query coverage and 0.0 E value.

The consensus sequence for the LSU region of *Hygrophorusscabrellus* was 763 bp. BLAST results revealed that these sequences were 96% similar to *Hygrophoruscossus* (AY548963 & KF381555) with 100% query coverage.

The *H.scabrellus*LSU sequences clustered with high bootstrap support with similar taxa in the section Hygrophorus of subgenus Hygrophorus (Fig. 6). In both our LSU and ITS analyses, *H.scabrellus* formed a sister lineage to *H.cossus* from Sweden with strong bootstrap support (Figs 5, 6).

*Hygrophorusalboflavescens* basidiomata were collected from Shawar Valley. Its ECM roots were collected from the same valley. The species falls into *Hygrophorus*, subgenusColorati, section Pudorini and subsectionClitocyboides. *Hygrophorusscabrellus* clusters within subsect. Hygrophorus, section Hygrophorus of subgenus Hygrophorus. Some of the ECM root sequences clustered with *H.pudorinus* sequences (FJ845408) from Canada and *H.pudorinus* (KT875016) from Mexico in the phylogenetic tree (Fig. 5). *H.pudorinus* belongs to Subgenus *Colorati*, section *Pudorinii* 2, subsection *Pudorini*. The collection of this ECM is the first report of this species from Pakistan.

### Taxonomy

#### Hygrophorus alboflavescens

Taxon classificationFungiAgaricalesHygrophoraceae

A. Naseer & A.N. Khalid sp. nov.

MB828146

Figures 1 , 2

##### Diagnosis.

*Hygrophorusalboflavescens* can be distinguished from related species by its white, centrally depressed pileus having yellow dots, with straight, even margins; occurrence of white stipe with yellow patches at lower half and broader (4.98 μm) basidiospores.

**Figure 1.**
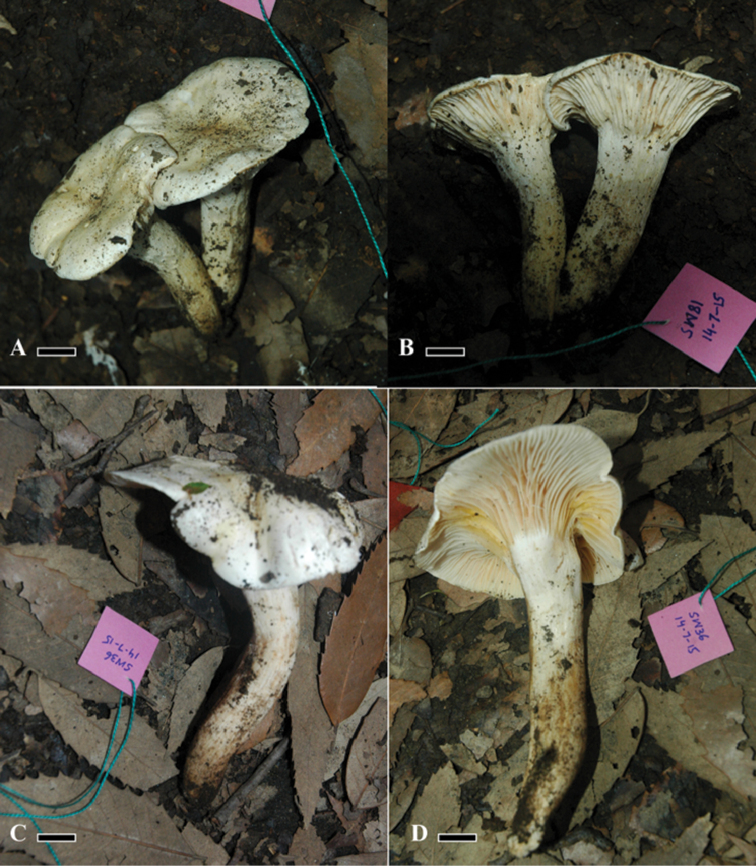
Morphology of *Hygrophorusalboflavescens* (Holotype). **A–D** Basidiomata **A, B**LAH35244; FLAS-F-59457 **C, D**LAH35243. Scale bar: 1.5 cm.

**Figure 2.**
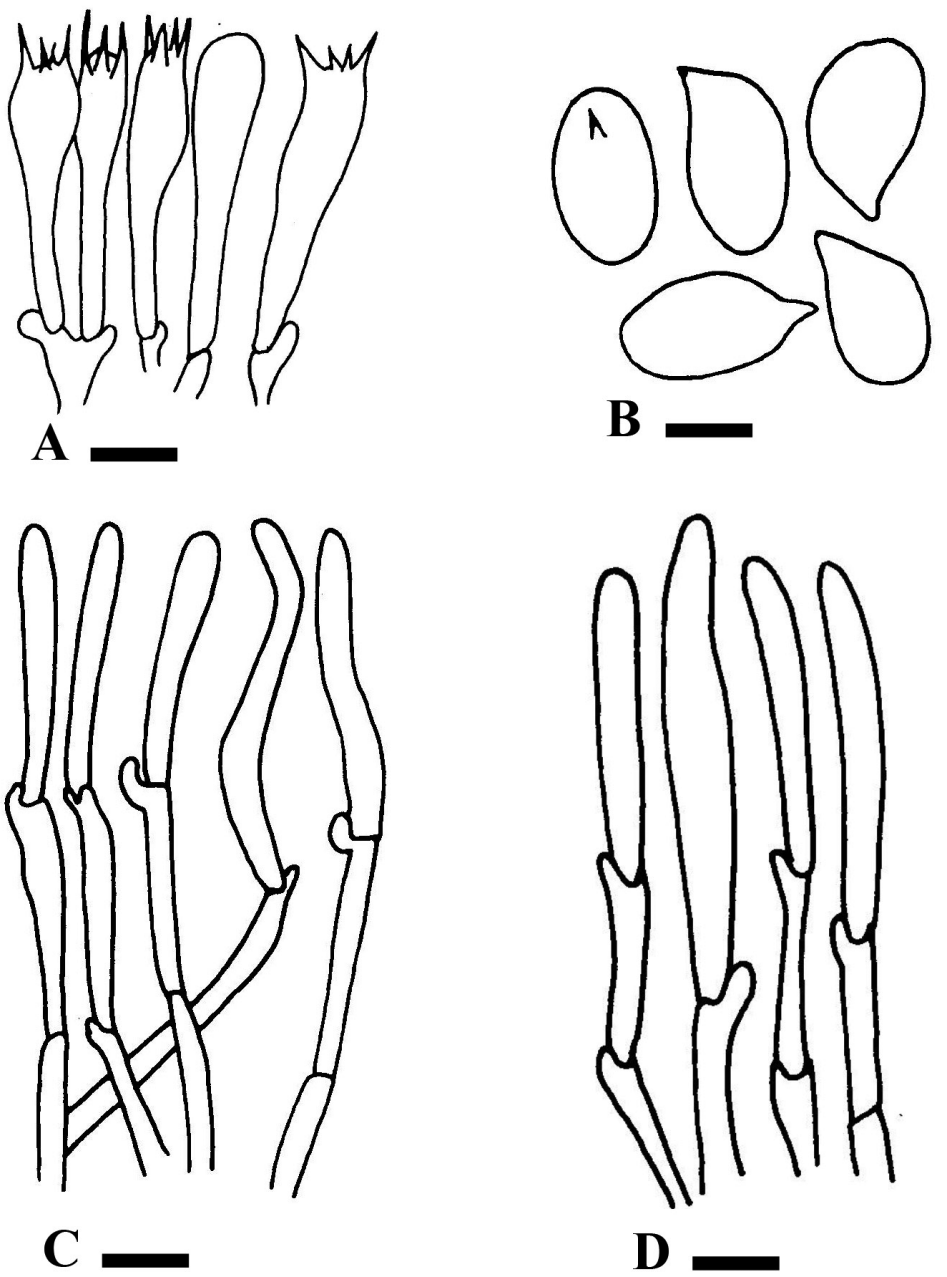
Anatomy of *Hygrophorusalboflavescens*. **A–D**LAH35243 (holotype) **A** Basida **B** Basidiospores **C** Pileipellis **D** Stipitipellis. Scale bars: 2.0 μm (**A**); 4.5 μm (**B**); 13.7 μm (**C**); 7.8 μm (**D**).

##### Typification.

PAKISTAN. Khyber Pakhtunkhwa Province, Swat, Shawar Valley, 2100 m alt., solitary or in pairs, on soil under *Quercusincana*, 14 July 2014, Arooj Naseer & Abdul Nasir Khalid, ASSW36 (holotype: LAH35243).

##### Etymology.

The species epithet refers to the white pileus with yellow dots and white stipe with yellow patches.

***Basidiomata*** medium to large sized. ***Pileus*** 7–10.5 cm in diameter, butter white (0.1B 8.8/0.3) with yellow (5.2Y 4.3/4) dots, plane, centrally depressed, context moderately thick, margin, even, smooth, straight, sometime incurved. ***Lamellae*** white (5.1GY 7.9/1.9) with yellow (6.1 Y 6.8/5.5) and pink (2.8Y 6.9/3.9) colouration, decurrent, thick, distant, L = 30–41, even, entire. ***Lamellulae*** irregular, of variable length, alternating with lamellae. ***Stipe*** 1.5–2.5 cm thick at apex, 0.5–1.5 cm at base, 8 –12.5 cm long, white (0.1B 8.8/0.3) with yellow (5.4Y 5.3/4) patches at lower half, cylindrical, slightly tapering at base, central, hollow.

***Basidiospores*** [60/3/2] (5.52–) 5.6–7.9 (–8.1) × (3.84–) 3.9–6.5 (–6.7), avL × avW = 6.64 × 4.98, Q = (1.20–) 1.21 × 1.40 (–1.43), avQ = 1.34, light green to hyaline in 5% KOH, ellipsoid, oblong, thick-walled. ***Basidia*** 31.6–48.8 × 5.8–6.7 μm, hyaline in 5% KOH, four-spored, clavate with long sterigmata (up to 3.0–4.2 μm), densely guttulated. ***Hymenophoral Trama*** 4–5.2 μm in diameter, thin-walled, branched, septate, oil contents, clamp connection present. ***Pileipellis*** an ixocutis of wide, thick hyphae, 3.0–5.5 μm in diameter. ***Stipitipellis*** a cutis of parallel and erect hyphae, 3.1–5.3 μm in diameter, light yellow in 5% KOH, septate. ***Clamp Connections*** present in all tissues.

##### Habit and distribution.

Solitary and in pairs on soil under *Quercusincana*, at 2100 m a.s.l., in thick moist temperate forest of the western Himalaya.

##### Additional material examined.

PAKISTAN, Khyber Pakhtunkhwa province, Swat, Shawar Valley, 2100 m a.s.l., solitary or in a pair, on soil under *Quercusincana*, 14 July 2014, Arooj Naseer & Abdul Nasir Khalid, ASSW81 (LAH35244; FLAS-F-59457).

##### Notes.

*Hygrophorusalboflavescens* nom. prov. can be distinguished from closely related species by the following combination of characters: a white, plane, centrally depressed pileus having straight margins; stipe that is white above and yellow below; and broadly ellipsoid spores. The closely related species *Hygrophoruspenarioides* is also an oak-specific species (Table 1). However, they differ morphologically. *Hygrophoruspenarioides* can easily be distinguished by its convex pileus with broad umbo and its involute margins (Jacobsson and Larsson 2007), whereas *H.alboflavescens* has centrally depressed pileus (without umbo) and straight margins. *Hygrophoruspenarioides* has a pure white pileus and stipe which become cream or slightly pinkish with age, whereas *H.alboflavescens* has a white stipe and pileus with yellow colouration on both. *Hygrophorusalboflavescens* has a longer stipe (8–12.5 cm) and broader spores (3.9–6.7 μm) as compared to *H.penarioides*. *Hygrophorusalboflavescens* is further differentiated from closely related taxa, *H.sordidus*, which has a convex, expanded to plane pileus that is larger (8–20 cm broad) compared with the smaller (7–10.5 cm broad), centrally depressed pileus of *H.alboflavescens. Hygrophorusalboflavescens* has even, smooth and straight margins that differ from involute and subnoccose margins of *H.sordidus*. Molecular analyses based on ITS and LSU regions also support *H.alboflavescens* as a distinct taxon and demonstrate its ECM relationship with oak in Pakistan.

**Table 1.** Comparsion of *Hygrophorus* spp. from Pakistan with morphologically similar species.

Characters/ Species	*H.alboflavescens* sp. nov.	*H.penarioides* Jacobsson & E. Larss.	*H.sordidus* Peck	*H.scabrellus* sp. nov.	*H.eburneus* (Bull.) Fr.	*H.cossus* (Sowerby) Fr.
**Pileus**						
Shape	Centrally depressed	Convex	Convex, expand to plane	Plano convex	Obtuse to convex	Broadly convex to nearly plane
Colour	Pure white with yellow dots	Pure white with creamy centre	Pure white or rarely tinged yellowish buff	Off-white with dark green	White	Pale orchraceous grey
Size	7–10.5 cm	9–15 cm	8–20 cm	2.4–2.8 cm	2–7(10) cm	3–7 cm
Umbo	No umbo	Broad umbo	No umbo	No Umbo	Umbonate	Obtuse nearly plane
Margins	Even, smooth, straight, sometime incurved	Strongly involute	Involute and subnoccose	Even, smooth, incurved	Even, involute and floccose-pubescent	Incurved
**Stipe**						
Surface	Dry, yellow patches on lower half	Finely floccose in uppermost part	Dry, glabrous, upper portion obscurely noccose	Scales on whole stipe	Fine scales at apex only and rest of stipe is smooth	Fibrillose-punctate to scabrous at apex, lower two-thirds covered by gelatinous sheath
Shape	Cylindrical	Strongly attenuated towards base	Equal, sometimes attenuated towards base	Cylindrical, finely scaled	Equal/tapered downward/ with a greatly attenuated vermiform base,	Equal, tapered at base
Colour	White with yellow patches at lower half	White, in lower part creamy	White	Yellowish-green with white apex	White stipe	Salmon-buff to cinnamon
Size	1.5–2.5 cm thick 8–12.5 cm long	15–35 mm thick 60–100 mm long	1.5–3.0 cm thick 6–10 cm long	0.3–0.5 cm thick 2.1–2.4 cm long	2–8(15) mm thick 4.5–15(18) cm long	(3)8–12 mm thick 4–9 cm long
**Basidiospores**						
Size	6.64 × 4.98 μm	1.13–1.6 μm	6–8 × (3.5) 4–5.5 μm	6.5 × 3.84 μm	6–8(9) × 3.5–5 μm	7–9 × 4–4.5 μm
Shape	Ellipsoid, oblong	Broadly ellipsoid to ovoid	Ellipsoid, smooth	Ovoid to ellipsoid	Ellipsoid, smooth	Ellipsoid
**Habitat**	Oak specific	Oak specific	Oak-hickory woods	Oak specific	*Fagus* specific	Oak specific

#### Hygrophorus scabrellus

Taxon classificationFungiAgaricalesHygrophoraceae

A. Naseer & A.N. Khalid sp. nov.

MB828147

Figures 3 , 4

##### Diagnosis.

*Hygrophorusscabrellus* is characterised by off-white, plano-convex pileus with greyish, dark green fibrils; yellowish-green, longer (2.1–2.4 cm) stipe with white apex and fine scales along the whole stipe; ovoid to ellipsoid, smooth and smaller (6.5 × 3.8 μm) basidiospores.

**Figure 3.**
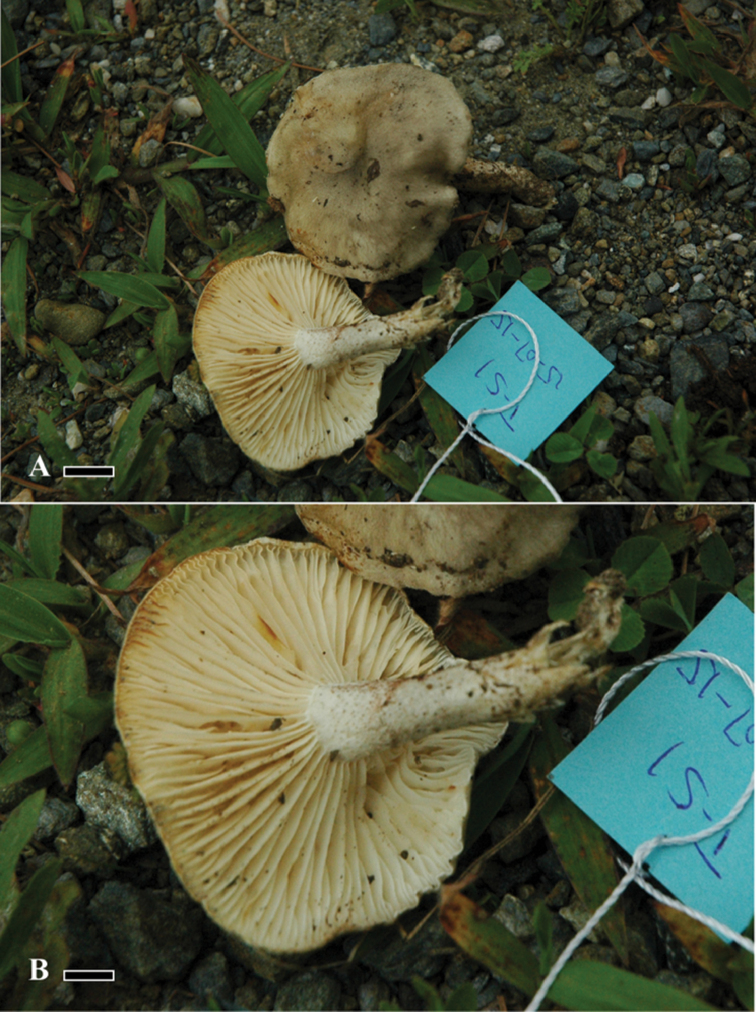
Morphology of *Hygrophorusscabrellus*. **A, B** Basidiomata. LAH35245 (holotype). Scale bars: 0.88 cm (**A**); 0.48 cm (**B**).

**Figure 4.**
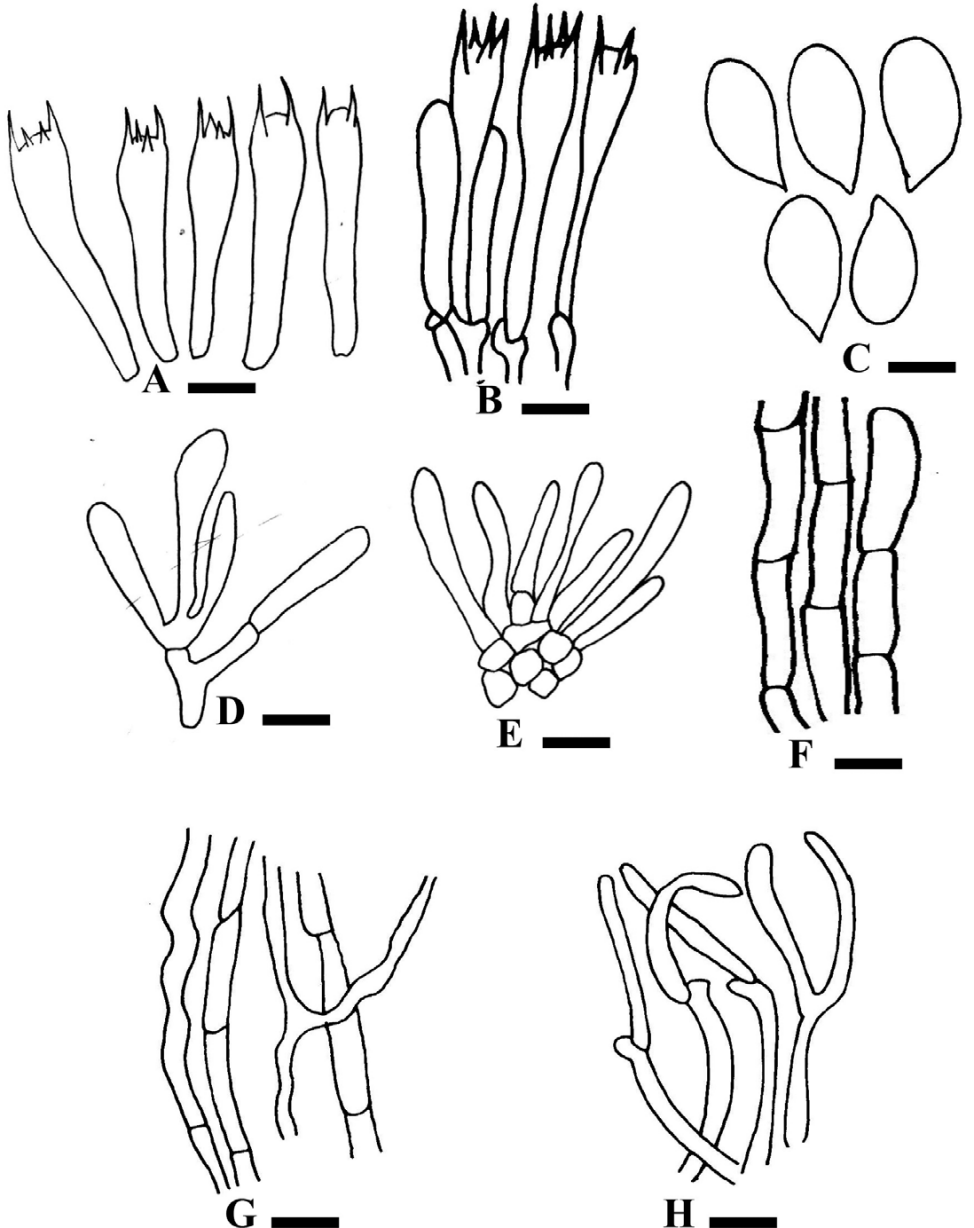
Anatomy of *Hygrophorusscabrellus*. **A–H**LAH35245 (holotype) **A** Basidia **B** Basidia with basidioles **C** Basidiospores **D** Cheilocystidia **E** Pleurocystidia **F** Stipitipellis **G** Tramal Hyphae **H** Pileipellis. Scale bars: 5.83 μm (**A, B, D, E**); 3.55 μm (**C**); 0.12 μm (**F–H**).

##### Typification.

PAKISTAN. Khyber Pakhtunkhwa Province, Swat, Toa, 2800 m a.s.l., on soil under *Quercusincana*, 15 July 2015, Arooj Naseer & Abdul Nasir Khalid, AST51 (holotype: LAH35245).

**Figure 5.**
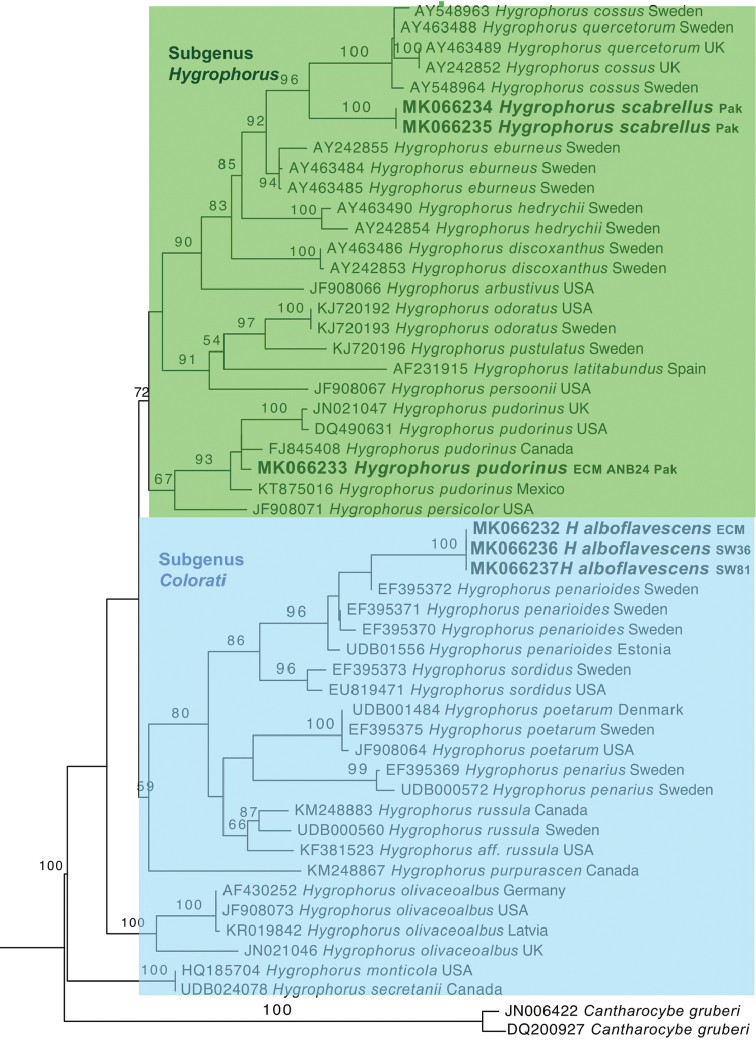
Phylogenetic relationship of *Hygrophorus* spp. and its ECM roots from Pakistan and their allied *Hygrophorus* species based on nrDNA ITS sequences using the Maximum Likelihood method. Sequences generated during this study are in bold letters. Sequences from root tips were labelled as ECM.

**Figure 6.**
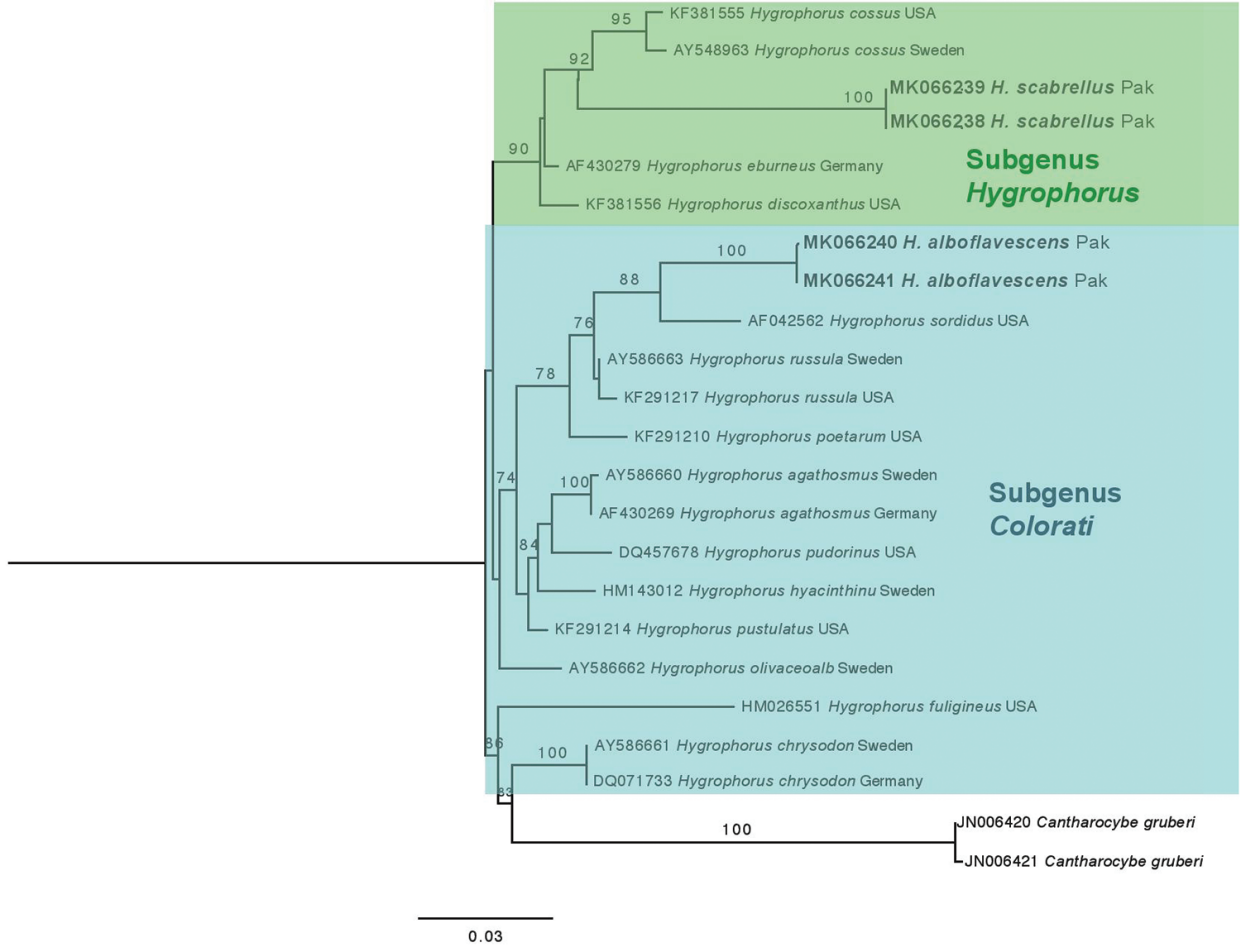
Molecular phylogenetic analysis of *Hygrophorus* spp. based on LSU sequences. Maximum likelihood phylogram of *Hygrophorus* based on nrDNA LSU as generated with RAxML with 1000 bootstrap iterations. Bolded lettering refers to sequences generated in this study.

##### Etymology.

The species epithet refers to the fine scales on the stipe.

***Basidiomata*** medium sized. ***Pileus*** 2.4–2.8 cm, creamy, off-white (7.9GY 6/1) with dark green, greyish fibrils (2.9GY 2.4/2), plano-convex, context moderately thick, margin even, smooth, incurved. ***Lamellae*** off-white to beige (4GY 6.8/2.4), subdecurrent to decurrent, thick, spaced to moderately close, L= 41–49, even, entire, undulate at margins. ***Lamellulae*** short, in two tiers, 1/3 of length of lamellae. ***Stipe*** 2.1–2.4 cm long, 0.3–0.5 cm in diameter, yellowish-green (9.3Y 4.4/2.4) with white (6.9GY 7/1) apex, finely scaled, cylindrical, slightly tapering at base, hollow.

***Basidiospores*** [30/1/1] (4.56–) 4.72–8.1 (–8.76) × (2.5–) 2.8–5.1 (–5.2) μm, avL × avW = 6.5 × 3.84 μm, Q = (1.5–) 1.57 × 1.89 (–1.86), avQ = 1.70, white to light yellow in 5% KOH, ovoid to ellipsoid, smooth, inamyloid. ***Basidia*** 30.2–42.3 × 6.8–9.3 μm, hyaline to light green in 5% KOH, narrowly clavate, four-spored, sterigmata long (6.2–7.2 μm), medium thick-walled, densely guttulate. ***Hymenophoral Trama*** 3.7–8.2 μm in diameter, bilateral, divergent hyphae, thin-walled, branched, septate. ***Pileipellis*** 3–3.7 μm in diameter, an ixotrichoderm, composed of branched septate hyphae. ***Stipitipellis*** 3.2–7.0 μm, a thin ixocutis to ixotrichoderm, composed of compact erect hyphae. ***Clamp Connections*** present in all tissues.

##### Habitat and distribution.

Solitary on soil under *Q.incana*, at 2800 m a.s.l., in moist temperate forest of Hindu Kush Himalayan range.

### Comments

*Hygrophorusscabrellus* is characterised by a yellowish-green stipe with a white apex that has fine scales on the entire stipe, planoconvex pileus which is off-white with dark green and greyish fibrils.

*Hygrophorusscabrellus* differs morphologically from the phylogenetically related species *H.eburneus*. *Hygrophoruseburneus* has fine scales only at the stipe apex (Table 1), whereas *H.scabrellus* has scales along the entire length of the stipe. *Hygrophoruseburneus* has a white stipe (yellowish-green stipe with a white apex in *H.scabrellus*). *Hygrophoruseburneus* also differs in having a pure white cap. Our new species *H.scabrellus* is similar to *Hygrophoruscossus* (Sow. ex Berk.) Fr. commonly known as Goat Moth Wax Cap, as both share plano-convex pileus. However, *H.cossus* has greyish white, broader pileus (3–9 cm) and smaller stipe (0.6–2 cm long) (Larsson and Jacobsson 2004) as compared to *H.scabrellus* that is distinguished by off-white pileus with dark green and greyish fibrils (2.4–2.8 cm) having longer stipe (2.1–2.4 cm). Anatomically, *H.cossus* has larger basidiospores (7–9 × 4–5 μm) (Larsson and Jacobsson 2004). Molecular phylogenetic analyses based on ITS and LSU sequences also support *Hygrophorusscabrellus* as a distinct species with strong bootstrap support.

## Discussion

In this paper, two new species of *Hygrophorus* were studied morphologically and sequences of two DNA regions were analysed for each species. These studies revealed that *H.alboflavescens* falls into section PudoriniofsubgenusColorati and differs from other species in the section by having yellow dots or patches rather than having entirely white basdiomata. We also confirmed, based on ITS sequences from roots, that this new species forms ECM associations with *Q.incana*. *Hygrophorusscabrellus* clusters within section Hygrophorus of subgenus Hygrophorus and differs in colour and stipe scaliness from others in that subgenus. These two new species provide evidence that further research is needed to collect and identify the fungal diversity of Asia, which appears to be a global hotspot of fungal diversity.

## Supplementary Material

XML Treatment for Hygrophorus alboflavescens

XML Treatment for Hygrophorus scabrellus

## Supplementary materials

Supplementary material 1 Microscopic features of *Hygrophorusalboflavescens* Data type: media Explanation note: **A–E**LAH35243 (holotype). **A** Pleurocystidia; **B** Basidaia with Cheilocystidia **C, D** Basidiopsores; **E** Pileipellis. This dataset is made available under the Open Database License (http://opendatacommons.org/licenses/odbl/1.0/). The Open Database License (ODbL) is a license agreement intended to allow users to freely share, modify, and use this Dataset while maintaining this same freedom for others, provided that the original source and author(s) are credited.Arooj Naseer, Abdul Nasir Khalid, Rosanne Healy, Matthew E. Smith

Supplementary material 2 Microscopic features of *Hygrophorusscabrellus* Data type: media Explanation note: **A–D**LAH35245 (holotype). **A** Basidiospores; **B** Hyphal Trama; **C** Basidiospores; **D** Pileipellis. This dataset is made available under the Open Database License (http://opendatacommons.org/licenses/odbl/1.0/). The Open Database License (ODbL) is a license agreement intended to allow users to freely share, modify, and use this Dataset while maintaining this same freedom for others, provided that the original source and author(s) are credited.Arooj Naseer, Abdul Nasir Khalid, Rosanne Healy, Matthew E. Smith
