# Supplementary figures and images for: Two new species of Hygrophorus from temperate Himalayan Oak forests of Pakistan
Source: MycoKeys. 2019 Jul 10;56:33–47. doi: 10.3897/mycokeys.56.30280 (PMC6637032; doi:10.3897/mycokeys.56.30280)

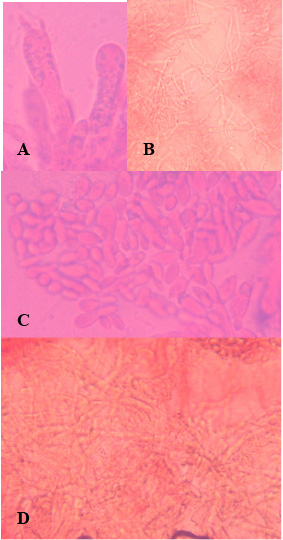

Supplement: Supplementary material 1 [file mycokeys-56-033-s002.jpg]
